# Potential life years not saved due to lack of access to anti-EGFR tyrosine kinase inhibitors for lung cancer treatment in the Brazilian public healthcare system: Budget impact and strategies to improve access. A pharmacoeconomic study

**DOI:** 10.1590/1516-3180.2018.0256170919

**Published:** 2020-03-06

**Authors:** Pedro Aguiar, Carmelia Maria Noia Barreto, Felipe Roitberg, Gilberto Lopes, Auro del Giglio

**Affiliations:** I MD, MSc. Physician and Consultant, Department of Oncology, Faculdade de Medicina do ABC (FMABC), Santo André (SP), and Physician and Consultant, Américas Centro de Oncologia Integrado, São Paulo (SP), Brazil.; II MD. Fellow, MD Anderson Cancer Center, Houston (TX), United States.; III MD. Physician and Consultant, Instituto do Câncer do Estado de São Paulo (ICESP), São Paulo (SP), Brazil.; IV MD, FAMS, MBA. Physician, Head of Global Oncology, Sylvester Comprehensive Cancer Center, University of Miami, Miami, USA.; V MD, PhD. Physician and Professor, Centro de Estudos em Hematologia e Oncologia, Faculdade de Medicina do ABC, Santo André (SP), Brazil.

**Keywords:** Health policy, Molecular targeted therapy, Economics, pharmaceutical, Gefitinib, Afatinib, Erlotinib, Non-small cell lung cancer, Access to therapy, Budget impact assessment

## Abstract

**BACKGROUND:**

Lung cancer is the fourth most common cancer in Brazil. In the 2000s, better understanding of molecular pathways led to development of epidermal growth factor receptor (EGFR)-targeted treatments that have improved outcomes. However, these treatments are unavailable in most Brazilian public healthcare services (Sistema Único de Saúde, SUS).

**OBJECTIVE:**

To assess the potential number of years of life not saved, the budget impact of the treatment and strategies to improve access.

**DESIGN AND SETTING:**

Pharmacoeconomic study assessing the potential societal and economic impact of adopting EGFR-targeted therapy within SUS.

**METHODS:**

We estimated the number of cases eligible for treatment, using epidemiological data from the National Cancer Institute. We used data from a single meta-analysis and from the Lung Cancer Mutation Consortium (LCMC) study as the basis for assessing differences in patients’ survival between use of targeted therapy and use of chemotherapy. The costs of targeted treatment were based on the national reference and were compared with the amount reimbursed for chemotherapy through SUS.

**RESULTS:**

There was no life-year gain with EGFR-targeted therapy in the single meta-analysis (hazard ratio, HR, 1.01). The LCMC showed that 1,556 potential life-years were not saved annually. We estimated that the annual budget impact was 125 million Brazilian reais (BRL) with erlotinib, 48 million BRL with gefitinib and 52 million BRL with afatinib. Their incremental costs over chemotherapy per life-year saved were 80,329 BRL, 31,011 BRL and 33,225 BRL, respectively. A drug acquisition discount may decrease the budget impact by 30% (with a 20% discount). A fixed cost of 1,000 BRL may decrease the budget impact by 95%.

**CONCLUSION:**

Reducing drug acquisition costs may improve access to EGFR-targeted therapy for lung cancer.

## INTRODUCTION

Over recent decades, the incidence of cancer and the mortality that it causes have increased in developing countries such as Brazil.^1^ Among all neoplasms, lung cancer presents major concern because it is a relatively frequent disease (i.e. the fourth most common type of cancer in Brazil) and presents high lethality. In Brazil, 28,220 new cases were expected in 2017; almost 25,000 were recorded in 2013.^2^

Studies on the molecular biology of advanced non-small cell lung cancer (NSCLC) have led to development of directed targeted therapies that have demonstrated better clinical outcomes and fewer collateral effects, compared with platinum-doublet chemotherapy.^3^^,^^4^^,^^5^

Epidermal growth factor receptor (EGFR) is a transmembrane receptor that conducts signals to promote cell proliferation, angiogenesis and cell immortality.^6^ Treatment of advanced NSCLC with tyrosine kinase inhibitors (TKI) directed against the EGFR receptor leads to a response rate of over 50% (that of chemotherapy is approximately 35%), and a nearly 100% increase in median progression-free survival, compared with platinum-doublet chemotherapy.^3^^,^^4^^,^^5^ On the other hand, no studies have demonstrated any increase in overall survival, compared with platinum-doublet chemotherapy, due to the high rate (about 70%) of treatment crossover between the arms of these studies. In other words, the majority of individuals included in such clinical studies received anti-EGFR TKI in first-line or second-line treatment.^3^^,^^4^^,^^5^

A Cochrane Library meta-analysis confirmed that anti-EGFR TKI was beneficial in relation to chemotherapy, in terms of response rate and progression-free survival. However, there was no improvement in terms of overall survival for any molecule.^7^

Although the Brazilian Ministry of Health considers that anti-EGFR TKI is a therapeutic option in cases of advanced lung cancer with the presence of EGFR mutation, the reimbursement offered by the Ministry of Health is insufficient for targeted therapy to be used. Consequently, the most common treatment among Brazilian public healthcare services is platinum-doublet chemotherapy, which does not provide median overall survival surpassing 12 months.^8^^,^^9^

Anti-EGFR TKIs cost as much as two to six times more than the 1,100.00 Brazilian reais (BRL) that are reimbursed by the Brazilian public healthcare system (Sistema Único de Saúde, SUS) for each month of treatment for metastatic lung cancer.^10^^,^^11^ The progressive increase in the cost of cancer treatment is a growing concern worldwide. In Brazil, data from the Federal Court of Auditors show that the annual cost of cancer treatment within SUS doubled between 2002 and 2008, from about 250 million to 500 million BRL.^12^ This increase in the costs of cancer treatment has outpaced the increase in average family income and inflation.^13^ Consequently, the budget impact caused by cancer treatment can make healthcare systems unsustainable, thus provoking user coverage failures.^14^ Consequently, choosing the best treatment at an accessible cost is a growing challenge for both clinics and managers.^15^

In view of this scenario, providers are placed in a difficult position with regard to acquiring anti-EGFR TKIs with reimbursement from the Ministry of Health. Consequently, there has been a loss of potential life-years that could have been saved through personalized treatment. Furthermore, several strategies can reduce the budget impact caused through incorporation of such medication, thereby making these agents available for treating SUS patients.

## OBJECTIVE

The aim of this study was to assess the potential number of years of life not saved, the budget impact of the treatment and strategies to improve access.

## METHODS

### Estimation of the number of eligible patients

The number of patients eligible for treatment was calculated using the number of new cases estimated for Brazil from 2010 (the year in which the first anti-EGFR TKI was launched in Brazil) to 2017. The estimated number of new cases was published by INCA (the National Cancer Institute of Brazil).^2^ The proportion of patients with the disease at an advanced stage and who were in a clinical condition to be able to receive first-line treatment was estimated based on the 2014 National Lung Cancer Audit in the United Kingdom and the European study of real-world treatment data published by Moro-Sibilot et al.^16^^,^^17^ The proportion of patients with activation of mutations in the EGFR gene was extracted from the largest database available in the literature.^18^

### Clinical benefits of treatment

We evaluated the clinical benefit of targeted therapy by calculating the number of life-years saved, compared with chemotherapy. This was based on the hazard ratio retrieved from a single meta-analysis.^19^ We also considered the areas under the overall survival curves of the American population study, Lung Cancer Mutation Consortium, since there are no data on the Brazilian population. The Lung Cancer Mutation Consortium study included 14 US centers and prospectively evaluated the overall survival of patients with metastatic NSCLC who either had or had not undergone molecular alterations appropriate for personalized treatment, and who either had or had not received targeted therapy directed against previously detected molecular alterations.^20^ We considered a five-year timeline, which was estimated in accordance with the overall survival curve of the Lung Cancer Mutation Consortium study.

### Costs of treatments and of the EGFR test

The Brazilian reference costs of acquisition of anti-EGFR TKI were considered.^10^ On the other hand, treatment with chemotherapy was considered to have a monthly fixed cost of 1,100.00 BRL, which is the amount reimbursed through SUS.

The costs of the EGFR mutation test (Sanger DNA sequencing) were considered for analysis, even though this test is available at no cost from the pharmaceutical industry.

The median duration of each treatment was based on the area under the progression-free survival curve, as published in randomized clinical trials.^3^^,^^4^^,^^5^^,^^21^

The costs of treating adverse events, drug infusion, hospitalization and support care were extrapolated from Brazilian data available in the literature.^22^^,^^23^^,^^24^

To better understand the total potential economic impact of each treatment strategy, we assumed that the hypothetical market penetration of anti-EGFR TKIs after their release was 100%.

### Endpoints

The primary objectives of this study were to evaluate the annual incremental cost (in millions of BRL) of incorporating anti-EGFR TKIs into routine use within SUS and the number of potential life-years not saved due to the unavailability of treatment using this medication within SUS since the time when the first EGFR TKI agent came into the market in 2010.

We also estimated the impact on the budget of the following strategies: cost sharing (giving the first two months of treatment at no charge), risk sharing (the corresponding manufacturer would reimburse 50% of the cost to non-responders), payment according to results (reimbursement of 100% of the cost in cases of progressive disease) and discounts (10% or 20%). Strategically, we also evaluated a hypothetical scenario in which the anti-EGFR TKI had a fixed cost of 1,000.00 BRL, i.e. a hypothetical value below the Brazilian Ministry of Health reimbursement level.

The secondary objectives of this study were to estimate the incremental cost for each year of life saved through using each anti-EGFR TKI and the additional cost per citizen of incorporation of each treatment, assuming a Brazilian population of 200 million inhabitants.

### RESULTS

### Estimated number of eligible cases

In 2017, the estimated number of patients with advanced or metastatic NSCLC was 20,261 in Brazil. Out of this total, a little more than half (57.4%) satisfied the clinical conditions for receiving any first-line treatment; and about one in four (25.5%) presented activation of mutations in the EGFR gene. Considering that 75% of the Brazilian population is treated through SUS, 2,224 cases were estimated to be eligible to receive anti-EGFR TKI in 2017.

### Budget impact

The annual investment necessary for incorporating anti-EGFR TKIs into SUS was estimated to be 125.1 million BRL for erlotinib, 48.3 million BRL for gefitinib and 51.7 BRL for afatinib. These amounts represent proportional increases, compared with the current SUS costs of acquiring antineoplastic agents, of 5.2%, 2.0% and 2.2%, respectively. The incremental costs per SUS user for incorporating these treatments were estimated to be 0.83 BRL, 0.32 BRL and 0.34 BRL, respectively.

### Cost sharing

The cost-sharing strategy, in which the first two months of treatment would be provided by the manufacturer of the medication, led to a 20% reduction in incremental cost. Through this strategy, the investments to incorporate erlotinib, gefitinib or afatinib to the SUS would be 100.2 million BRL, 36.2 million BRL or 39.1 million BRL, respectively.

### Risk sharing and payment according to results

The risk-sharing strategy would reduce the annual budget impact by 25 million BRL for use of erlotinib, 9 million BRL for gefitinib and 16 million BRL for afatinib. These amounts represent reductions of 27%, 17% and 32%, respectively.

Payment according to results presented more modest results, with a reduction in the annual incremental cost of erlotinib, gefitinib and afatinib of approximately 4 million BRL for each drug. Proportionally, payment according to results gave rise to reductions of 2%, 7% and 9%, respectively.

### Discounts and fixed cost at 1,000.00 BRL

With a 10% reduction in the cost of anti-EGFR TKIs, the annual incremental cost of their incorporation into SUS was reduced by 12% for use of erlotinib, 15% for gefitinib and 14% for afatinib.

A 20% discount resulted in annual savings of 30 million BRL for use of erlotinib and 15 million BRL for gefitinib and afatinib each. These amounts represent reductions of 24%, 30% and 29%, respectively.

The greatest reduction in the budget impact of the treatment was observed through setting the cost of anti-EGFR TKIs at 1,000.00 BRL. Through this strategy, it would be possible to save more than 90% of the funds required to incorporate the NSCLC targeted therapy into SUS. The annual incremental costs of erlotinib, gefitinib and afatinib would be 2.88 million BRL, 2.9 million BRL and 3.0 million BRL, respectively. These values represent an additional investment of 0.1%, compared with the current cost to SUS of acquiring antineoplastic drugs. The incremental cost per user of this strategy is 0.02 BRL.

**Figure 1.**
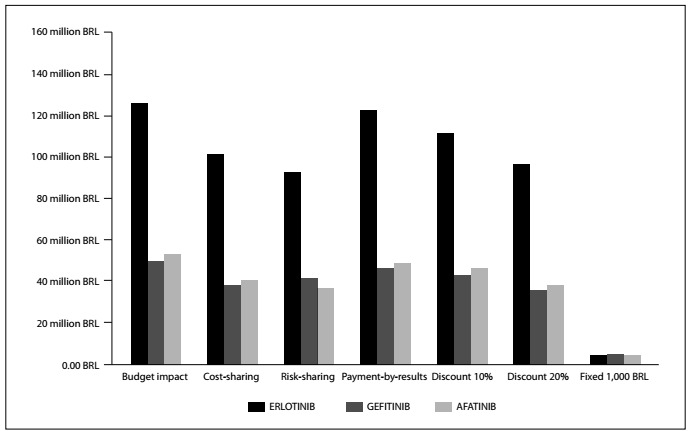
Budget impact in the base case and with strategies to reduce the incremental cost. Amounts in Brazilian reais (BRL).

Figure 1 summarizes the incremental costs of incorporating anti-EFGR TKIs at baseline, and when using the various strategies presented.

### Potential life-years not saved

The data from the single meta-analysis did not show any difference in overall survival for patients treated with anti-EGFR TKI, in comparison with those receiving chemotherapy (hazard ratio 1.01, 95% CI 0.88 to 1.17, P = 0.84). Considering the data from the Lung Cancer Mutation Consortium, the lack of access to targeted anti-EGFR therapy within SUS has resulted in around 1,556 potential life-years not saved annually. Over the last seven years since the launch of gefitinib in Brazil, 10,892 potential life-years have not been saved.

### Cost-effectiveness

The data from the Lung Cancer Mutation Consortium study show that the strategy of offering molecular targeted therapy to eligible patients resulted in 0.70 life-years saved in comparison with chemotherapy alone.

In the base case, the average incremental cost per patient treated with erlotinib was 56,230.33 BRL, resulting in an incremental cost per year of life saved of 80,329.05 BRL. Gefitinib had an incremental cost per patient of 21,707.77 BRL, thus resulting in 31,011.10 BRL per life-year saved. Use of afatinib increased the cost per patient by 23,257.88 BRL, and the cost per life-year saved was 33,225.55 BRL. Table 1 summarizes the costs considered and the cost-effectiveness findings.

**Table 1.** Estimated costs and cost-effectiveness, in Brazilian reais (BRL)

	Erlotinib	Gefitinib	Afatinib
Price	5,581.55	2,701.94	2,824.43
Cost of treatment	66,978.60	32,423.28	33,893.16
Cost of adverse events	54,74	87,50	167,74
Cost of monitoring	5,384.64	5,384.64	5,384.64
Additional life-years	0.70	0.70	0.70
Incremental cost	56,230.33	21,707.77	23,257.88
Cost per additional life-year	80,329.05	31,011.10	33,225.55

## DISCUSSION

Historically, cancer patients have had few therapeutic options and a poor prognosis. However, oncology has advanced over recent decades, especially in terms of secondary prevention, either through early detection of disease or through development of new therapies that have increased patient survival.^25^ Although these advances are to be commended, they have been made at a high cost financially, sometimes making them inaccessible for developing countries.

Between 2010 and 2014, 25 new drugs were approved for cancer treatment in the United States.^26^ This figure represented half of all the new medications that had been approved over the previous four decades.^26^ Access to new cancer treatments in developing countries is made more complicated through the fact that these new therapies generally cost more than the reference drugs and are administered over longer periods of time.^26^

One major goal of doctors and public healthcare administrators in Brazil is to ensure that resources for acquiring antineoplastic agents are allocated efficiently. Cost-effectiveness studies are the most widely used tool for establishing the value of a treatment, considering the effectiveness of the drug and its direct and indirect costs.^15^

Several Brazilian studies have evaluated whether anti-EGFR TKIs are cost-effective for treating lung cancer.^22^^,^^23^^,^^24^ The study that defined the SUS policy was conducted by the National Commission on Incorporation of Technologies in SUS (CONITEC). It concluded that targeted therapy was not cost-effective, mainly based on the absence of an overall gain in life-years survived.^22^ However, the CONITEC study relied on data from a randomized clinical study in which most individuals included were exposed to targeted therapy at some phase of treatment (first-line or second-line). However, the crossover rate between the treatment arms was nearly 70%.

Interestingly, despite the lack of overall survival benefit, anti-EGFR TKI can improve quality-adjusted life-years (QALY), compared with chemotherapy.^27^ A previous study by our group found a gain of 0.18 QALY, considering the data from the single meta-analysis, and a gain of 0.50 QALY considering the Lung Cancer Mutation Consortium data.^27^ In terms of cost-effectiveness, the incremental costs per QALY (ICER) were 30,000 BRL and 70,000 BRL, respectively.^27^ Curiously, the lower ICER observed in the single meta-analysis, compared with the Lung Cancer Mutation Consortium, was due to the crossover between the arms. In other words, everybody received TKI and consequently the same amount of money was spent on every case.

The consequence of a situation in which a given treatment is unavailable through the healthcare system is an increase in “judicialization” of healthcare. This practice, even if it does democratize access to new therapies, also raises costs and ultimately engenders more inequality.^28^ Over a recent five-year period, the Brazilian federal government’s expenditure on medicines obtained through court orders increased by 517%, from 42.8 million BRL in 2010 to 259.4 million BRL in 2014.^28^

We believe that measures to facilitate universal access to innovative medicines can reduce the costs associated with lawsuits and allow healthcare systems to negotiate prices with the industry through obtaining volume discounts. In our study, we found that the practice of offering discounts for acquiring medicines was an effective strategy, as this reduced the budget impact by 15% and 30%, for discounts of 10% and 20%, respectively. Setting the cost of medication below the amount currently reimbursed through SUS for chemotherapy reduced the budget impact by more than 90%, thus making it possible for the SUS investment in incorporating these agents to remain below inflation (a 0.1% increase in the cost of acquisition of antineoplastic agents).

Other strategies to reduce the budget impact of a treatment include cost sharing and risk sharing. In our study, compared with obtaining discounts, cost sharing and risk sharing presented only modest benefits in relation to acquiring drugs. Our hypothesis to explain the failure of cost sharing relates to the fact that patients receive treatment for an average period of 12 months, which makes receiving the initial months without cost proportionally less relevant.

The high rate of disease control (around 90%) achieved through anti-EFGR TKIs makes risk sharing a poor option for decreasing the budget impact. Furthermore, Brazil does not have laws or regulatory mechanisms that would permit implementation of a strategy of shared risks. The Italian experience has shown that systems for managing risk sharing have significant costs (almost one million euros annually), and up to one third of cases are not reimbursed by manufacturers because of administrative issues like errors or delays in completing reimbursement forms.^29^

The main limitation of the present study lay in our attempt to make estimates for the Brazilian population based on potentially underestimated epidemiological data, and on clinical data that was extrapolated from a study on the United States population. Ideally, a prospective randomized study on the Brazilian population, comparing anti-EGFR TKI for EGFR-mutated advanced NSCLC patients with platinum-doublet chemotherapy for patients who do not undergo the molecular test, should be conducted. However, a study with this design would not be authorized by any research ethics committee, given that the benefits of using targeted molecular therapy have already been established in the literature.

## CONCLUSIONS

We believe that targeted anti-EGFR therapy for metastatic NSCLC is a cost-effective treatment in terms of cost per life-year saved. An investment of 2% of the amount paid for acquiring antineoplastic agents, or additional expenditure of around 0.30 BRL per user, could improve patient access to anti-EGFR therapy. Moreover, negotiation of discounts with manufacturers or implementation of a price control policy can reduce the budget impact by 90%. Improving the access of SUS patients to anti-EGFR therapy could potentially save more than 1,500 life-years annually.
